# Genetic Diversity and Reproduction Trends of *Phytophthora infestans* in Estonia: EU_41_A2 Detected without an Indication of Clonal Reproduction

**DOI:** 10.3390/jof10030233

**Published:** 2024-03-21

**Authors:** Britt Puidet, Mati Koppel, Riinu Kiiker

**Affiliations:** 1 Institute of Agricultural and Environmental Sciences, Estonian University of Life Sciences, Kreutzwaldi 1, 51006 Tartu, Estonia; mati.koppel@emu.ee; 2 Centre of Estonian Rural Research and Knowledge, 48309 Jõgeva, Estonia; riinu.kiiker@metk.agri.ee

**Keywords:** genetic diversity, pathogen population monitoring, *Phytophthora infestans*, population dynamics, Simple Sequence Repeat (SSR) genotyping

## Abstract

This study explores the population dynamics of *Phytophthora infestans* in Estonia from 2005 to 2022, focusing on genetic diversity and potential shifts in reproductive strategies. In total, 153 *P. infestans* isolates were collected throughout Estonia over ten growing seasons. Genotyping revealed considerable genetic diversity, with most isolates not corresponding to known multilocus genotypes (MLGs). Still, instances of invasive clonal lineages were observed, notably EU_41_A2. The data indicate the likelihood of random mating rather than clonal reproduction in all the analyzed years. The principal coordinate analysis (PCoA) results revealed no distinct clustering among the sampling years. Statistical analysis and the minimum spanning network (MSN) indicated low genetic differentiation between years with minimal fluctuations in allele frequencies. The continuous monitoring of *P. infestans* populations is essential for detecting any changes from the current evolutionary trajectory and implement effective disease management strategies, especially considering the recent emergence of EU_41_A2 in the Nordics and the potential impacts of climate change.

## 1. Introduction

*Phytophthora infestans*, an oomycete pathogen, has caused devastating epidemics, severely impacting potato and tomato production globally. The pathogen’s ability to rapidly adapt and overcome resistance measures [1,2] requires a comprehensive understanding of the pathogen’s population dynamics. In Northern Europe, the pathogen’s populations have been characterized by a wide range of genetic diversity and probable sexual reproduction events [3,4,5]. Epidemics have been initiated by soil-borne oospores, which survive the cold winters [5].

Recently, there has been a noticeable shift in these populations. While genetically diverse isolates remain predominant, new genotypes like EU_41_A2 and EU_43_A2 have become established in the region [6,7,8]. This transition might signify that *P. infestans* populations in Northern Europe have experienced changes in their reproductive strategies, raising questions about the factors responsible for this transition and its implications for the pathogen’s evolution and disease spread. Understanding the factors driving the transition from genetic diversity to clonal–sexual hybridization is important for implementing effective disease management strategies. Populations with sexual reproduction can be managed by targeting oospores through crop rotation, while populations with asexual reproduction can be managed by mitigating the risk of fungicide resistance development in clones due to the selection process.

The progression of a disease is a result of the combined influence of multiple elements that impact both the host and the pathogen. Even minor variations in microclimate conditions may alter the results of the interaction between the plant and the pathogen [9]. It is anticipated that *P. infestans* will be little affected by rising temperatures owing to its preference for cooler thermal conditions [10]. However, given the temperature increase observed in recent decades, *P. infestans* may acclimate, potentially leading to genetic adaptation and enhanced fitness [11].

Climate models suggest that global warming may lead to more frequent extreme temperatures at both ends of the spectrum [12]. Such temperature variability is likely to favor pathogen phenotypes with wider temperature niches. Consequently, the discovery that *P. infestans* strains with a larger thermal tolerance are also more resistant to pesticides is concerning. This is underscored by the observed correlation between azoxystrobin resistance and the temperature range in *P. infestans* [13].

Observations in Estonia have shown a delay in the beginning of epidemics or a total absence of late blight infection after prolonged heat and drought periods [14]. The resulting lower primary inoculum load may cause population bottlenecks or other ecological phenomena in the *P. infestans* populations. The loss of genetic diversity can alter the pathogen’s evolutionary trajectory and facilitate the establishment of clonal lineages [15]. However, it remains unclear whether shifts in climatic patterns have contributed to changes in pathogen population dynamics in Estonia.

Intensive agricultural practices, including the widespread cultivation of susceptible host varieties and the increased deployment of fungicides containing the same active ingredient, have the potential to exert selective pressure on *P. infestans* populations. Such practices may favor the emergence and spread of fungicide-resistant clonal lineages (e.g., EU_13_A2, EU_37_A2, EU_43_A1), thus influencing shifts in population structure and leading to disease control failures [7,16,17,18]. The virulence and fitness advantages of newly emerged clonal lineages (e.g., EU_41_A2) could play a significant role in their rapid expansion and subsequent persistence within the genetically diverse population [6].

Since its emergence in 2013, the genotype EU_41_A2 has rapidly spread across Northern Europe, reaching Norway and Sweden in 2016 and Finland in 2019 [9]. We hypothesized that EU_41_A2 might have also spread to Estonia and conducted a country-wide study. The purpose of this study was to understand any genetic changes that might enable this genotype to establish itself in Estonia. For this, we analyzed the potential genetic shifts within the population over the years.

## 2. Materials and Methods

### 2.1. Isolate Collection and Genotyping

**Figure 1:**
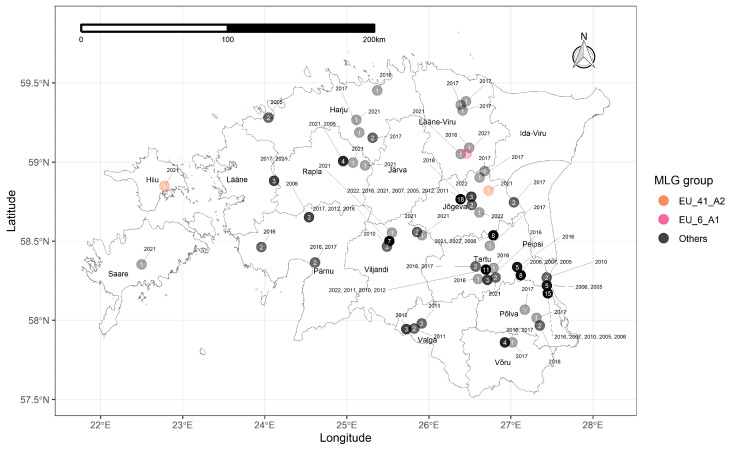
*Phytophthora infestans* isolates collected from Estonia by sampling year and multilocus genotype group (MLG). Each circle represents a sampling site, the color shade and the number in the circle correspond to the number of isolates collected. Base map from GADM 3.6 (https://gadm.org/download_country36.html, accessed on 20 March 2024).

In total, 153 *P. infestans* isolates were collected in ten growing seasons during a period of 18 years from 2005 to 2022 (Table 1). The isolates were collected throughout Estonia from conventional and organic fields, large- and small-scale growers, and untreated experimental fields (Figure 1). The research included 30 isolates from 2010 to 2012 from a previously published study [19]. Single *P. infestans* lesions were sampled from fields randomly from potato (*Solanum tuberosum* L.) leaves exhibiting typical late blight symptoms, each from a different plant. The samples were collected either on a Flinders Technology Associates (FTA) card (Whatman Classic FTA card, Cytiva, Marlborough, MA, USA) or as leaflets from where axenic cultures with distinct morphological characteristics were isolated.

The samples of *P. infestans* were isolated in the laboratory to rye B agar and grown at 18 °C in the dark. When the Petri dishes were fully colonized, the mycelia were collected into 2 mL tubes and placed at −80 °C for long-term storage and −20 °C for short-term storage until DNA extraction.

The DNA from mycelia was extracted using the DNeasy^®^ Plant Mini Kit (QIAGEN N.V., Hilden, Germany). The DNA and the FTA cards were sent to the James Hutton Institute, where the samples were genotyped with a 12-plex SSR marker set [20]. Genotypes were assigned to clonal lineages or regarded as unique by matching their SSR profiles to reference isolates in the Euroblight database [8].

### 2.2. Statistical Analysis

Data analysis for the SSR profiles for all 153 isolates was performed using the Microsoft Office Excel 2016 (Microsoft Corporation, Redmond, WA, USA) add-in GenAlEx (version 6.503), developed by Peakall, R. and Smouse, P.E. at the Australian National University, Canberra, Australia [21,22]. The add-in was used to calculate the loci’s allele frequency and gene diversity. The third allele in the triploid isolates had to be discarded to analyze the samples with the add-in, which was the case for less than 8% of all the samples in loci Pi02, Pi4B, G11, SSR4, SSR6, and SSR8.

The allele frequency was calculated as the proportion of the allele in a single year. The expected heterozygosity, i.e., gene diversity of a locus, was calculated using the following formula $H_{e}=1-\sum_{i=1}^{I}p_{i}^{2}$, where I is the number of distinct alleles at the locus and *p* is the frequency of the *i*th allele at the locus [23]. The genetic structure was analyzed with the inbreeding coefficient (F_IS_) and fixation index (F_ST_). F_IS_ was calculated by subtracting the observed heterozygosity (H_o_, the proportion of heterozygous samples at a given locus) from the expected heterozygosity averaged across years (H_e_) and dividing the value with H_e_. F_ST_ was calculated by subtracting H_e_ from the expected heterozygosity averaged for the overall total population (H_t_) and dividing the value with H_t_. Nei’s genetic distance [24] and F_ST_ values were analyzed through pairwise comparison among the sampled years. The significance of pairwise F_ST_ values was tested using 999 permutations. Genotypic diversity was calculated using a normalized Shannon’s diversity index (H_s_) [25].

The data analysis in R (R Core Team version 4.3.0, 2023) was continued with the 11 loci using the poppr package (version 2.9.4, Kamvar Z.N., Tabima J.F., Grünwald N.J., Australian National University, Canberra, Australia; Oregon State University, Corvallis, OR, USA) [26]. The extent of linkage disequilibrium among alleles at different loci within populations was calculated using the standardized association index (${\bar{r}}_{d}$). Genotypic diversity within each population, based on the number of unique multilocus genotypes (MLGs), was calculated using the Stoddart and Taylor’s index (G) [27]. The evenness of the distribution of MLGs in the population was calculated using the Evenness value (E.5). Genetic dissimilarity between individuals was calculated with Bruvo’s genetic distance [28]. The results were visualized using principal coordinate analysis (PCoA) to identify clusters of genetic similarity between the sampling years. To further explore the genetic relatedness among the *P. infestans* isolates, a minimum spanning network (MSN) analysis based on Bruvo’s genetic distances was performed.

## 3. Results

### 3.1. Allele Frequencies

The SSR profiles were analyzed for allele frequency (Table S1). Locus D13 had 34% of its data missing from the whole data set. As up to 54% of the data were missing for most of the sampling years, the locus D13 was omitted from the analysis. This corroborates with Kiiker et al.’s [4] finding that D13 is an uninformative locus for describing the Estonian population. Other loci (G11, Pi63, Pi70, SSR11, and SSR8), which had up to 14% missing data in some years, were left in the analysis.

A total of 54 alleles were found in the eleven loci, all of which were polymorphic (Table S1). The least number of alleles, only two, were found in locus SSR2. The most alleles were found in loci SSR4 and G11 (9 and 15, respectively). Still, even among the loci with many different alleles, only a few dominated most of the years (284 and 294 in SSR4; 154 and 162 in G11). About 40% of all different alleles (22 out of 54) had an overall frequency below 0.05. These alleles were found only on a few isolates in the same year. Also, they were quite rare throughout the years. Twelve alleles were found only in one year and, five were found in two out of the ten sampling years.

### 3.2. Genetic Diversity

Most of the isolates did not correspond to any of the MLGs in the EuroBlight database and were classified in the “others” group of isolates [8]. However, in 2021, one isolate from the mainland corresponded to genotype EU_6_A1, and two isolates (one from the mainland and one from the island of Hiiumaa) corresponded to genotype EU_41_A2 (Figure 1).

**Figure 2:**
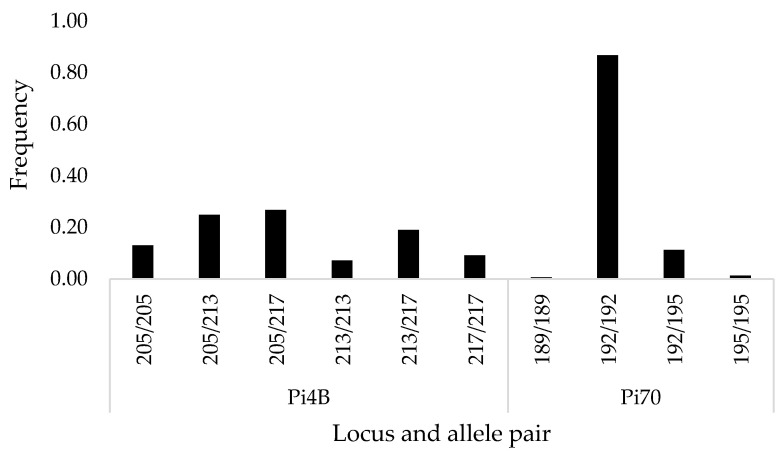
Allele pair frequencies of *Phytophthpra infestans* isolates sampled in Estonia (2005–2021) for two loci with three alleles—mainly homozygous Simple Sequence Repeat (SSR) locus Pi70 (*n* = 151) and highly heterozygous Pi4B (*n* = 153).

Gene diversity (H_e_) was calculated for each SSR locus (Table S1). The H_e_ value ranged from 0.142 for locus Pi70 to 0.786 for locus G11. The low H_e_ value shows low heterozygosity at locus Pi70, which has one dominant homozygous genotype (192/192 with a frequency of 0.87) (Figure 2). The highest H_e_ value was calculated for locus G11, where 31 different allele pairs were found, only 7 of which were homozygous. Still, the most frequent genotypes were homozygous (162/162 with a frequency of 0.21 and 154/154 with a frequency of 0.14). The analysis also showed high genetic diversity for locus Pi4B (H_e_ = 0.662), where, on the contrary, the most frequent genotypes were heterozygous (205/217, 205/213, and 213/217), and only six different genotypes were found (Figure 2).

Altogether, 104 different locus genotypes were found for the 11 analyzed loci (Table S2). All loci, except Pi4B, SSR11, SSR2, and SSR8, had allele pairs that occurred only once throughout all the years. In locus G11, there were 15 of such throughout all the monitored years. Interestingly, the F_IS_ for G11 was the highest of all loci (Table 2), which indicates fewer heterozygotes than expected. The high value of the inbreeding coefficient shows the locus is not in Hardy–Weinberg equilibrium, as is the case with locus Pi04, which has a high negative F_IS_ value, and locus Pi70, which has a high positive F_IS_ value. The F_ST_ values were between 0.024 and 0.071, which shows that there is very low genetic differentiation between the years (Table 2).

Combining alleles from the 11 SSR loci resulted in MLGs for all 153 isolates. A total of 119 were unique, making up 55% to 100% of the isolates collected in the same year (Table 1). Most of the MLGs found more than once were from the same locations in the same years. The maximum number of the same MLGs found more than once was three in 2006. H_s_ was high throughout all years, ranging from 0.822 in 2022 to 1.000 in 2016 (Table 1). Furthermore, the pairwise analysis of genetic differentiation (F_ST_ ranging from 0 to 0.038) and Nei’s genetic distance (ranging from 0 to 0.041) revealed a low level of genetic differentiation throughout the years (Table 3).

G, measuring the number of unique MLGs in relation to the sample size, showed high genotypic diversity across all years (Table 1). The highest G values were measured in 2016 and 2017, whereas the lowest appeared in 2022. The E.5 value, which assesses the evenness of the distribution of MLGs within the population each year, shows high evenness in most years (E.5 > 0.9). This indicates that the population had a relatively balanced distribution of genotypes and that there was no major dominance of any single genotype. The evenness in 2006 and 2022 was still relatively high but lower when compared to the rest of the population (E.5 < 0.9).

The data consistently indicate random mating for most of the evaluated years (Table 1). Even though the years 2011, 2021, and 2022 show negative ${\bar{r}}_{d}$ values, the null hypothesis of no linkage among the alleles within a population failed to be rejected (*p* > 0.001) and, therefore, supports the likelihood of random mating rather than clonal reproduction in these years.

**Figure 3:**
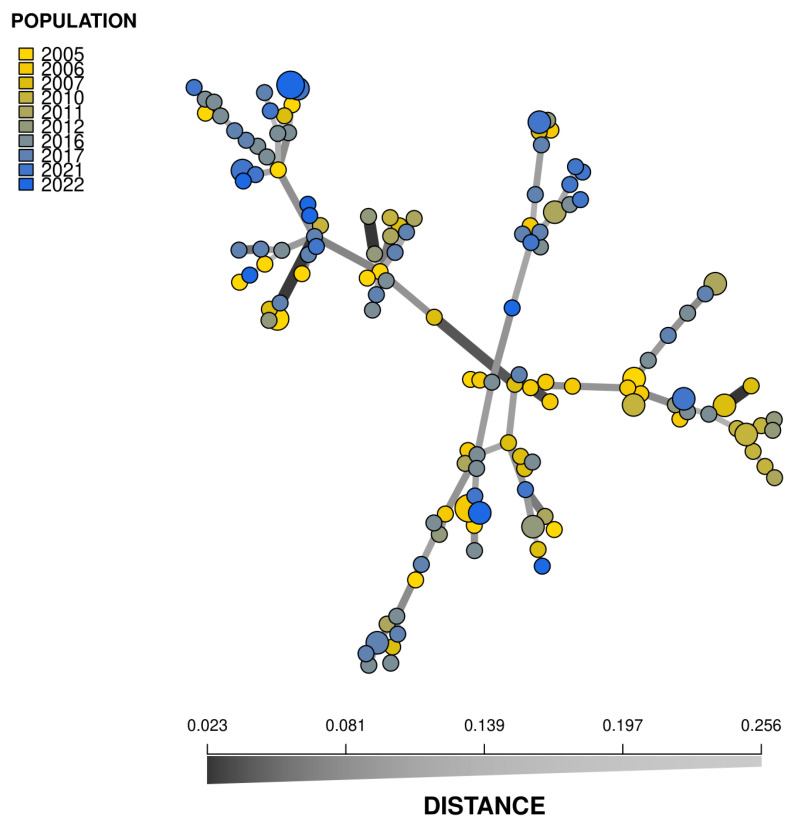
Minimum spanning network (MSN) based on Bruvo’s genetic distances of *Phytophthora infestans* isolates analyzed with 11 SSR markers. Each node represents one multilocus genotype (MLG). The node size corresponds to the number (1–3) of isolates with the same MLG, whereas the longer and lighter edges indicate a greater genetic distance between them.

**Figure 4:**
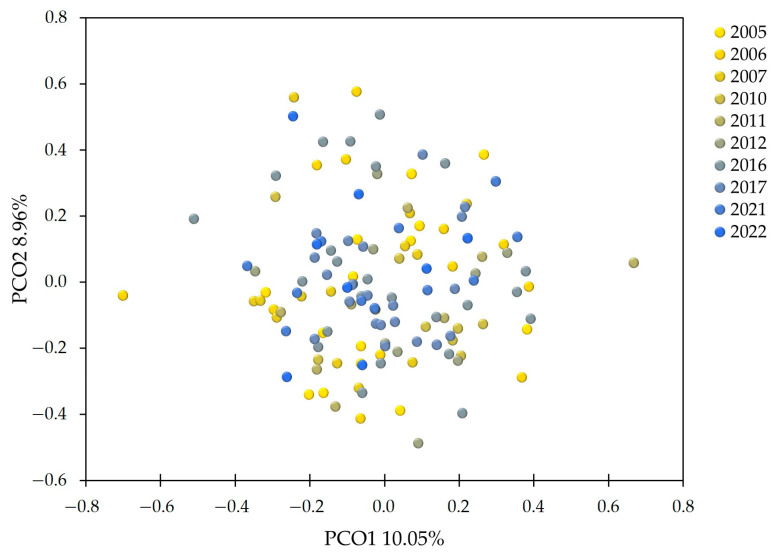
Principal coordinate analysis (PCoA) based on Bruvo’s genetic distances, illustrating the lack of clustering of Estonian *Phytophthora infestans* isolates by sampling year.

The MSN had several clades compiled of MLGs from different collecting years (Figure 3). MSN showed high relatedness between MLGs collected in consecutive years. In addition, the MSN revealed some closely related similar MLGs from 2005 to 2022. None of the MLGs were shared over the years. The PCoA results revealed no distinct clustering among the sampling years (Figure 4).

## 4. Discussion

Both *P. infestans* mating types have been present in Europe since the 1980s [29]. Henceforth, the production of oospores has become a relevant survival mechanism in the life cycle of *P. infestans* and a new primary source of inoculum in Northern Europe [5]. In Estonia, previous studies of *P. infestans* at the beginning of the 2000s showed a near-even presence of A1 and A2 mating types [30]. The oospore inoculum is increased by numerous small household potato growers who have limited availability for both, adequate crop protection and long crop rotation in their gardens [31,32].

The Estonian *P. infestans* population is genetically and genotypically highly diverse. During the study period of 2005 to 2022, a high percentage of MLGs was found in most years, and invasive clonal lineages were observed only on a few occasions. This is supported by G results, the high E.5 values, H_s_, and H_e_ analysis in this study. The results also correspond to previous studies conducted in this region [4,19].

This high genetic diversity and the results from the ${\bar{r}}_{d}$ suggest that the population reproduces sexually. The data indicate a rise in linkage disequilibrium in 2006, which means there was likely more clonal reproduction that year. Despite this, subsequent years did not show a continued trend towards clonality. This could imply that the factors favoring certain genotypes in 2006 were temporary, and these genotypes were outcompeted in the following years. No departure from random mating was proven, even in 2021, where three isolates with genotypes widely distributed in Europe (EU_6_A1 and EU_41_A2) were found.

The results of our study resemble those of Kiiker et al. [4], showing that Pi70 is the least informative and G11 is the most informative locus. While a range of alleles was identified at the G11 locus, a select few, notably alleles 154, 156, 160, and 162, were predominantly observed. These findings align with those of previous studies in the Nordics, Baltics, and Poland [19,33,34]. When a locus exhibits high heterozygosity, whereas most individuals primarily show homozygous genotypes, it suggests a complex dynamic within that population. The presence of many unique allele pairs alongside a high F_IS_ at the locus might suggest a selective pressure, inbreeding, or a population bottleneck. However, the results for genetic diversity suggest a somewhat stable or even increasing genetic diversity at different loci over time. Additionally, there is no drastic shift from one allele pair to another as the dominant one over the years.

The population’s genetic structure does not remain completely static, as evidenced by slight deviations from the Hardy–Weinberg equilibrium. But despite the changes, minimal genetic differentiation between the years suggests that while individuals might show variations, the broader population remains genetically consistent. The variation is attributed to the presence of sexual reproduction arising from the surviving oospores, the differences in the sampled fields, and the weather conditions of the years. This genetic consistency was also shown in the MSN analysis, as there were several clades of MLGs compiled from different collecting years, and there was a high degree of relatedness between the MLGs collected in consecutive years. Especially interesting is the occurrence of similar MLGs between the years 2005 and 2022, as it suggests that the population has remained relatively unchanged for a long period.

The establishment of EU_41_A2 in Denmark, Norway, and other Northern European countries raises multiple questions regarding the underlying ecological, genetic, and environmental factors [6]. The invasive success of EU_41_A2 may be attributable to the synergy of its inherent fitness characteristics and shifting environmental conditions that have become more favorable to the persistence of clones as a consequence of climate change [6]. As *P. infestans* exhibits adaptation to specific temperature ranges in different regions, the likelihood of the pathogen causing widespread outbreaks is based on prevailing local temperature conditions [35]. Therefore, the findings of clonal lineage EU_41_A2 in two different regions (island and mainland) in Estonia were not unexpected.

One plausible hypothesis for the findings of EU_41_A2 is that the bottleneck effect during the years with unfavorable weather conditions could have led to a loss in genetic variation. If, epidemiologically, there were years where the pathogen population was critically low due to adverse conditions, it might have allowed for specific genotypes or alleles which were previously rare or suppressed to be successful when the conditions became favorable again. The growing seasons of 2018 and 2019 were unfavorable for late blight development, with prolonged drought and high-temperature periods, and only very few late blight outbreaks were recorded [14]. Prolonged unfavorable conditions decreased the late blight pathogen population size, but an effect on diversity was not observed in our research. Instead, the detection of EU_41_A2 and EU_6_A1 in 2021 seems to be a random occurrence, and the clones were probably imported with the seed potatoes.

Although no similar large-scale sampling has been conducted since the EU_41_A2 was detected in Estonia in 2021, genotyping *P. infestans* in the same area on the mainland in the subsequent growing seasons [14] resulted in only unique MLGs. While the bottleneck effect during unfavorable years provides a compelling explanation for the reduction in the inoculum from volunteer tubers, the oospores in the soil were still able to withstand unfavorable weather conditions. Although the number of viable oospores also reduced in time [36], the population size might have been big enough and the genetic structure might have been stable enough for the bottleneck effect not to take place.

Specific local weather patterns and differences in agricultural practices or cultivated potato varieties could affect how the lineage spreads and adapts to new areas. While the climatic conditions in Estonia are similar to those of the Nordics, most of the cultivars grown in Estonia are susceptible to the pathogen, and a limited number of sprays are applied during the growing seasons. These factors do not support selection for higher virulence or for fungicide resistance among pathogen populations. The potato growing area is very small, declining from 13,959 ha in 2005 to only 3381 ha in 2022 [37]. Additionally, there are a lot of sparsely located small-scale farmers, which, in turn, reduces the selection pressure on the pathogen. Our research shows that infections in Estonian potato fields start from oospores in the fields of small-scale growers. Accordingly, control measures should be directed towards decreasing the formation and survival of infectious oospores, and thereby, the primary inoculum, through better disease control and prolonged crop rotations.

Previous epidemics of *P. infestans* have shown that the pathogen’s populations need to be monitored continuously [38]. This is well illustrated through the recent emergence of the mandipropamid-resistant genotype EU_43_A2 in the Danish population, which was detected in a timely manner through meticulous monitoring and well controlled by adequately applied measures [7,39]. Even though the establishment of clonal lineages in Estonia has not been proven, it is important to keep monitoring the population nationwide. The current study and previous studies [4,19] have shown a high diversity in Estonia, but even infrequent occurrences of sexual reproduction are enough to generate considerable variation within a population [40,41]. Moreover, it is suggested that single-nucleotide polymorphism (SNP) markers and genotyping-by-sequencing (GBS) might reveal a higher clonality of isolates than with SSR analysis [41,42]. Therefore, future studies should focus on other markers for a more comprehensive analysis of the population changes.

## 5. Conclusions

Our comprehensive study of *P. infestans* in Estonia from 2005 to 2022 reveals a complex and evolving pathogen population. The analysis shows that the population is genetically diverse, with a high percentage of unique MLGs. The study indicated that the method of reproduction is predominantly sexual, although instances of isolates from clonal lineages like EU_41_A2 were observed. The results show that the population has remained relatively unchanged for a long period, potentially due to the lack of selection pressure on the potato fields.

The detection of the clonal lineage EU_41_A2 in Estonia, paralleling its spread in other Northern European countries, underscores the need for continuous monitoring and research. While our study did not find conclusive evidence of a shift in pathogen reproduction strategy, the presence of EU_41_A2 and EU_6_A1 in 2021 suggests potential introductions through imported seed potatoes.

The genetic complexity and adaptability of *P. infestans* in Estonia call for a holistic approach to disease management, integrating genetic studies, local environmental conditions, and agricultural practices. Future research should employ advanced genotyping techniques to provide a more nuanced understanding of the pathogen’s population structure and dynamics in Estonia.

## Figures and Tables

**Table 1:** Characteristics of *Phytophthora infestans* collected from across Estonia from 2005 to 2022 by sampling year. Percentages of isolates with unique multilocus genotypes (MLGs), normalized Shannon’s diversity index (H_s_) values, standardized index of association (${\bar{r}}_{d}$) values, Stoddard and Taylor’s index (G) values, and Evenness (E.5) values are presented.

Year	Number of Isolates	Unique MLGs	H_s_	${\bar{r}}_{d}$	G	E.5
2005	16	75%	0.938	0.016	12.80	0.947
2006	15	80%	0.919	0.092 *	10.71	0.880
2007	14	86%	0.962	0.028	12.25	0.963
2010	10	60%	0.880	0.022	7.14	0.934
2011	10	60%	0.880	−0.014	7.14	0.934
2012	10	80%	0.940	0.041	8.33	0.952
2016	25	100%	1.000	0.003	25.00	1.000
2017	23	91%	0.981	0.014	21.16	0.976
2021	19	58%	0.901	−0.015	13.37	0.938
2022	11	55%	0.822	−0.022	6.37	0.868
All years	153	78%	0.966	0.003	121.29	0.939

* Calculated probability: *p* ≤ 0.001

**Table 2:** Inbreeding coefficient values within populations (F_IS_) and fixation index between populations (F_ST_) (2005 (*n* = 16), 2006 (*n* = 15), 2007 (*n* = 14), 2010 (*n* = 10), 2011 (*n* = 10), 2012 (*n* = 10), 2016 (*n* = 25), 2017 (*n* = 23), 2021 (*n* = 19), and 2022 (*n* = 11)) for 11 SSR loci from *Phytophthora infestans* isolates collected from Estonia.

Locus	F_IS_	F_ST_
Pi02	0.071	0.031
Pi4B	−0.082	0.045
G11	0.318	0.071
Pi04	−0.262	0.029
Pi63	−0.072	0.036
Pi70	0.140	0.065
SSR11	0.013	0.071
SSR2	−0.014	0.026
SSR4	−0.068	0.054
SSR6	−0.054	0.029
SSR8	−0.091	0.024

**Table 3:** *Phytophthora infestans* isolates’ genetic differentiation by fixation index (F_ST_) (above diagonal) and Nei’s genetic distance (below diagonal) values between different sampling years (2005 (*n* = 16), 2006 (*n* = 15), 2007 (*n* = 14), 2010 (*n* = 10), 2011 (*n* = 10), 2012 (*n* = 10), 2016 (*n* = 25), 2017 (*n* = 23), 2021 (*n* = 19), and 2022 (*n* = 11)) in Estonia.

Year	2005	2006	2007	2010	2011	2012	2016	2017	2021	2022
2005	-	0.036	0.029	0.020	0.000	0.006	0.002	0.005	0.018	0.014
2006	0.041	-	0.006	0.032	0.013	0.038	0.008	0.027	0.029	0.014
2007	0.038	0.000	-	0.038	0.011	0.012	0.012	0.014	0.013	0.012
2010	0.019	0.032	0.035	-	0.000	0.022	0.026	0.017	0.011	0.026
2011	0.000	0.012	0.008	0.000	-	0.000	0.003	0.004	0.000	0.000
2012	0.008	0.041	0.012	0.020	0.000	-	0.013	0.005	0.019	0.016
2016	0.004	0.008	0.010	0.029	0.004	0.015	-	0.000	0.024	0.021
2017	0.003	0.028	0.013	0.014	0.002	0.005	0.000	-	0.016	0.033
2021	0.028	0.032	0.015	0.008	0.000	0.024	0.030	0.022	-	0.007
2022	0.020	0.012	0.009	0.025	0.000	0.017	0.025	0.035	0.007	-
All years	-	0.036	0.029	0.020	0.000	0.006	0.002	0.005	0.018	0.014

## Data Availability

Data are contained within the article and Supplementary Materials.

## Supplementary Materials

The following supporting information can be downloaded at: https://www.mdpi.com/article/10.3390/jof10030233/s1, Table S1: Gene diversity (H_e_) in 11 SSR loci and allele frequencies for all 153 sampled isolates by sampling year (2005 (*n* = 16), 2006 (*n* = 15), 2007 (*n* = 14), 2010 (*n* = 10), 2011 (*n* = 10), 2012 (*n* = 10), 2016 (*n* = 25), 2017 (*n* = 23), 2021 (*n* = 19), and 2022 (*n* = 11)) in Estonia.; Table S2: The number of allele pairs found in the isolates of the Estonian *P. infestans* population in the different sampling years (2005 (*n* = 16), 2006 (*n* = 15), 2007 (*n* = 14), 2010 (*n* = 10), 2011 (*n* = 10), 2012 (*n* = 10), 2016 (*n* = 25), 2017 (*n* = 23), 2021 (*n* = 19), and 2022 (*n* = 11)).
