# Supplementary material for: Genetic Diversity and Reproduction Trends of Phytophthora infestans in Estonia: EU_41_A2 Detected without an Indication of Clonal Reproduction
Source: J Fungi (Basel). 2024 Mar 21;10(3):233. doi: 10.3390/jof10030233 (PMC10970722; doi:10.3390/jof10030233)
Supplement: Supplementary file 1 [file jof-10-00233-s001.zip › jof-2925972-supplementary.pdf]

Table S1. Gene diversity (H_e) in 11 SSR loci and allele frequencies for all the sampled 153 isolates by sampling years (2005 (n = 16), 2006 (n = 15), 2007 (n = 14), 2010 (n = 10), 2011 (n = 10), 2012 (n = 10), 2016 (n = 25), 2017 (n = 23), 2021 (n = 19) and 2022 (n = 11)) in Estonia.

SSR locus	Allele	Frequency in a year										Gene diversity (H_e)
		2005	2006	2007	2010	2011	2012	2016	2017	2021	2022	
	258	0.000	0.000	0.143	0.050	0.000	0.000	0.020	0.043	0.026	0.000	
Pi02 (n = 153)	266	0.188	0.133	0.143	0.000	0.100	0.200	0.200	0.130	0.132	0.136	0.295
	268	0.813	0.867	0.714	0.950	0.900	0.800	0.780	0.826	0.842	0.864	
	205	0.438	0.500	0.321	0.300	0.300	0.150	0.540	0.413	0.289	0.409	
Pi4B (n = 153)	213	0.125	0.300	0.357	0.350	0.300	0.400	0.200	0.370	0.395	0.136	0.662
	217	0.438	0.200	0.321	0.350	0.400	0.450	0.260	0.217	0.316	0.455	
	134	0.000	0.000	0.000	0.000	0.000	0.000	0.020	0.000	0.000	0.000	
	142	0.000	0.100	0.036	0.000	0.000	0.050	0.000	0.000	0.000	0.045	
	146	0.000	0.000	0.071	0.000	0.000	0.000	0.000	0.000	0.000	0.000	
	150	0.000	0.000	0.000	0.000	0.000	0.000	0.000	0.025	0.000	0.000	
	152	0.100	0.000	0.000	0.000	0.000	0.000	0.020	0.000	0.000	0.000	
	154	0.333	0.267	0.143	0.200	0.250	0.150	0.340	0.400	0.029	0.136	
	156	0.233	0.000	0.000	0.050	0.150	0.350	0.180	0.200	0.147	0.045	
G11 (n = 147)	158	0.000	0.067	0.000	0.000	0.000	0.000	0.000	0.000	0.000	0.045	0.786
	160	0.100	0.000	0.179	0.200	0.100	0.100	0.080	0.175	0.235	0.318	
	162	0.167	0.500	0.393	0.450	0.500	0.150	0.260	0.200	0.471	0.364	
	164	0.000	0.000	0.000	0.000	0.000	0.000	0.020	0.000	0.000	0.000	
	166	0.033	0.000	0.000	0.000	0.000	0.000	0.000	0.000	0.000	0.000	
	206	0.033	0.067	0.179	0.100	0.000	0.200	0.040	0.000	0.059	0.045	
	208	0.000	0.000	0.000	0.000	0.000	0.000	0.020	0.000	0.000	0.000	
	210	0.000	0.000	0.000	0.000	0.000	0.000	0.020	0.000	0.059	0.000	

SSR locus	Allele	Frequency in a year										Gene diversity (H_e)
		**2005**	**2006**	**2007**	**2010**	**2011**	**2012**	**2016**	**2017**	**2021**	**2022**	
Pi04 (n = 153)	160	0.000	0.000	0.000	0.000	0.000	0.000	0.020	0.000	0.000	0.000	0.613
	166	0.344	0.467	0.464	0.400	0.400	0.300	0.440	0.391	0.421	0.182	
	168	0.125	0.000	0.107	0.100	0.200	0.200	0.080	0.152	0.211	0.364	
	170	0.531	0.533	0.429	0.500	0.400	0.500	0.460	0.457	0.368	0.455	
Pi63 (n = 151)	270	0.438	0.133	0.125	0.100	0.200	0.250	0.240	0.239	0.263	0.227	0.596
	273	0.125	0.333	0.333	0.250	0.150	0.150	0.160	0.130	0.263	0.318	
	276	0.000	0.033	0.000	0.000	0.000	0.000	0.000	0.000	0.000	0.000	
	279	0.438	0.500	0.542	0.650	0.650	0.600	0.600	0.630	0.474	0.455	
Pi70 (n = 151)	189	0.063	0.000	0.000	0.000	0.000	0.000	0.000	0.000	0.000	0.000	0.142
	192	0.781	0.967	0.929	1.000	0.850	1.000	0.958	0.978	0.842	0.950	
	195	0.156	0.033	0.071	0.000	0.150	0.000	0.042	0.022	0.158	0.050	
SSR11 (n = 150)	331	0.063	0.133	0.077	0.000	0.150	0.300	0.180	0.119	0.079	0.045	0.584
	341	0.594	0.367	0.346	0.800	0.650	0.450	0.400	0.548	0.658	0.500	
	355	0.344	0.500	0.577	0.200	0.200	0.250	0.420	0.333	0.263	0.455	
SSR2 (n = 153)	173	0.688	0.900	0.679	0.750	0.700	0.700	0.660	0.696	0.684	0.818	0.404
	175	0.313	0.100	0.321	0.250	0.300	0.300	0.340	0.304	0.316	0.182	
SSR4 (n = 151)	284	0.250	0.400	0.393	0.100	0.350	0.350	0.220	0.227	0.389	0.500	0.782
	286	0.000	0.000	0.036	0.000	0.000	0.000	0.000	0.023	0.000	0.000	
	288	0.156	0.100	0.250	0.100	0.100	0.100	0.200	0.250	0.111	0.045	
	290	0.000	0.000	0.000	0.000	0.000	0.000	0.040	0.023	0.139	0.000	
	292	0.250	0.200	0.071	0.300	0.050	0.100	0.260	0.250	0.222	0.091	
	294	0.313	0.267	0.179	0.400	0.400	0.400	0.240	0.136	0.028	0.273	
	296	0.031	0.033	0.036	0.100	0.100	0.050	0.040	0.091	0.083	0.000	
	298	0.000	0.000	0.036	0.000	0.000	0.000	0.000	0.000	0.028	0.000	

SSR locus	Allele	Frequency in a year										Gene diversity (H_e)
		2005	2006	2007	2010	2011	2012	2016	2017	2021	2022	
SSR6 (n = 153)	238	0.000	0.000	0.000	0.000	0.000	0.000	0.000	0.000	0.026	0.000	0.498
	240	0.000	0.000	0.036	0.000	0.000	0.100	0.000	0.000	0.026	0.045	
	242	0.375	0.400	0.214	0.500	0.400	0.250	0.400	0.283	0.447	0.455	
	244	0.625	0.600	0.679	0.500	0.600	0.650	0.600	0.717	0.500	0.500	
	246	0.000	0.000	0.071	0.000	0.000	0.000	0.000	0.000	0.000	0.000	
SSR8 (n = 147)	260	0.667	0.433	0.542	0.700	0.550	0.600	0.480	0.614	0.529	0.500	0.517
	264	0.033	0.100	0.000	0.000	0.000	0.000	0.020	0.023	0.059	0.000	
	266	0.300	0.467	0.458	0.300	0.450	0.400	0.500	0.364	0.412	0.500	

Table S2. The number of allele pairs found in the isolates of the Estonian *P. infestans* population in different sampling years (2005 (n = 16), 2006 (n = 15), 2007 (n = 14), 2010 (n = 10), 2011 (n = 10), 2012 (n = 10), 2016 (n = 25), 2017 (n = 23), 2021 (n = 19) and 2022 (n = 11)).

SSR locus	Allele pair	Number of isolates	Number of isolates in a year										Overall frequency
			2005	2006	2007	2010	2011	2012	2016	2017	2021	2022	
Pi02 (n = 153)	258/258	1			1								0.007
	258/266	3							1	1	1		0.020
	258/268	4			2	1				1			0.026
	266/266	4	1						2			1	0.026
	266/268	33	4	4	4		2	4	5	5	4	1	0.216
	268/268	108	11	11	7	9	8	6	17	16	14	9	0.706
Pi4B (n = 153)	205/205	20	1	3	1	2	1		6	2	2	2	0.131
	205/213	38	4	6	5		1	3	6	10	2	1	0.248
	205/217	41	8	3	2	2	3		9	5	5	4	0.268
	213/213	11		1	1	1	2			2	3	1	0.072
	213/217	29		1	3	5	1	5	4	3	7		0.190
	217/217	14	3	1	2		2	2		1		3	0.092

SSR locus	Allele pair	Number of isolates	Number of isolates in a year										Overall frequency
			2005	2006	2007	2010	2011	2012	2016	2017	2021	2022	
	134/160	1							1				0.007
	142/142	1		1									0.007
	142/156	1						1					0.007
	142/160	1			1								0.007
	142/162	1										1	0.007
	142/206	1		1									0.007
	146/146	1			1								0.007
	150/154	1								1			0.007
	152/152	1	1										0.007
	152/156	1	1										0.007
	152/162	1							1				0.007
	154/154	21	4	2	1	2	2		5	5			0.143
	154/156	7	1				1		2	3			0.048
	154/158	3		2								1	0.020
	154/160	5							2	1	1	1	0.034
G11 (n = 147)	154/162	9	1	2	2			1	1	1		1	0.061
	154/164	1							1				0.007
	154/206	3						2	1				0.020
	156/156	10	2				1	3	2	2			0.068
	156/162	5				1			1	1	1	1	0.034
	156/206	4	1						1		2		0.027
	156/208	1							1				0.007
	156/210	2									2		0.014
	160/160	8	1					1		3	3		0.054
	160/162	15	1		3	2	2		1		1	5	0.102
	160/206	4			1	2						1	0.027
	162/162	31	1	6	2	3	4	1	4	3	7		0.211
	162/166	1	1										0.007
	162/206	3		1	2								0.020
	162/210	1							1				0.007
	206/206	2			1			1					0.014

SSR locus	Allele pair	Number of isolates	Number of isolates in a year										Overall frequency
			2005	2006	2007	2010	2011	2012	2016	2017	2021	2022	
Pi04 (n = 153)	160/166	1							1				0.007
	166/166	1									1		0.007
	166/168	1			1								0.007
	166/170	116	11	14	12	8	8	6	21	18	14	4	0.758
	168/168	21	2		1	1	2	2	2	3	4	4	0.137
	168/170	1								1			0.007
	170/170	12	3	1		1		2	1	1		3	0.078
Pi63 (n = 151)	270/270	7	4						1		1	1	0.046
	270/273	16		3	2		2	1	2	1	2	3	0.106
	270/279	40	6	1	1	2	2	4	8	10	6		0.265
	273/273	6	1	3							2		0.040
	273/279	36	2	1	6	5	1	2	6	5	4	4	0.238
	276/279	1		1									0.007
	279/279	45	3	6	3	3	5	3	8	7	4	3	0.298
Pi70 (n = 151)	189/189	1	1										0.007
	192/192	131	12	14	12	10	7	10	22	22	13	9	0.868
	192/195	17	1	1	2		3		2	1	6	1	0.113
	195/195	2	2										0.013
SSR11 (n = 150)	331/331	2							2				0.013
	331/341	22	2	4			1	5	1	5	3	1	0.147
	331/355	9			2		2	1	4				0.060
	341/341	43	4	3	3	7	5	1	7	4	8	1	0.287
	341/355	48	9	1	3	2	2	2	5	10	6	8	0.320
	355/355	26	1	7	5	1		1	6	2	2	1	0.173
SSR2 (n = 153)	173/173	79	8	12	8	5	6	4	10	10	9	7	0.516
	173/175	62	6	3	3	5	2	6	13	12	8	4	0.405
	175/175	12	2		3		2		2	1	2		0.078

SSR locus	Allele pair	Number of isolates	Number of isolates in a year										Overall frequency
			2005	2006	2007	2010	2011	2012	2016	2017	2021	2022	
	284/284	13	2		3		1	2	2		1	2	0.086
	284/288	15	1	3	3		2			2	4		0.099
	284/290	4									4		0.026
	284/292	21	1	3			1	1	4	5	4	2	0.139
	284/294	20	2	5	2	1	2	2	2	1		3	0.132
	284/296	5		1		1			1	2			0.033
	284/302	2										2	0.013
	286/292	1								1			0.007
	286/298	1			1								0.007
	288/288	2							2				0.013
SSR4 (n = 151)	288/290	3							2	1			0.020
	288/292	4			1					3			0.026
	288/294	19	4		2	2		1	4	5		1	0.126
	288/296	2			1			1					0.013
	290/292	1									1		0.007
	292/292	6	2			2			2				0.040
	292/294	15	3	3	1	1		1	5		1		0.099
	292/296	4				1				2	1		0.026
	292/298	1									1		0.007
	294/294	7				2	2	2				1	0.046
	294/296	4	1				2		1				0.026
	296/296	1									1		0.007
	238/242	1									1		0.007
	240/242	3			1			1				1	0.020
	240/244	2						1			1		0.013
SSR6 (n = 153)	242/242	21	4	4	1	1			6	1	3	1	0.137
	242/244	67	4	4	3	8	8	4	8	11	10	7	0.438
	244/244	58	8	7	8	1	2	4	11	11	4	2	0.379
	246/246	1			1								0.007

SSR locus	Allele pair	Number of isolates	Number of isolates in a year										Overall frequency
			2005	2006	2007	2010	2011	2012	2016	2017	2021	2022	
SSR8 (n = 147)	260/260	43	7	3	2	4	4	3	4	9	4	3	0.293
	260/264	4	1						1		2		0.027
	260/266	73	5	7	9	6	3	6	15	9	8	5	0.497
	264/266	4		3						1			0.027
	266/266	23	2	2	1		3	1	5	3	3	3	0.156
